# Contemporaneous Presentation of Ocular Myasthenia Gravis With Pituitary Apoplexy: A Diagnostic Dilemma

**DOI:** 10.7759/cureus.80666

**Published:** 2025-03-16

**Authors:** Isuru Perera, Hiruni Fernando, KVC Janaka, Dimithri Gamaarachchi, Harsha Rathnayake

**Affiliations:** 1 Internal Medicine, Sri Jayawardenepura General Hospital, Colombo, LKA

**Keywords:** acute onset headache, bilateral ptosis, medial rectus palsy, myasthenia gravis (mg), occulomotor nerve palsy, ocular myasthenia gravis, pituitary apoplexy

## Abstract

Pituitary apoplexy is a life-threatening condition caused by a rapid expansion of the pituitary tumor due to hemorrhage or infarction. It usually presents with acute onset severe headache and can also be associated with visual field defects and ophthalmoplegia. Similarly, ocular myasthenia gravis, which is an autoimmune condition causing muscle fatiguability, also presents with ophthalmoplegia, commonly ptosis and diplopia. A 53-year-old male patient with a past history of adrenal insufficiency presented with acute onset headache. On examination, he had bilateral asymmetrical partial ptosis and left-side medial rectus palsy with mild fatiguability. He had normal visual fields and sparing of the pupils. Acetylcholine receptor antibodies were positive but failed to demonstrate a decremental response in nerve conduction studies. The patient was started on neostigmine on clinical suspicion of ocular myasthenia gravis. A magnetic resonance imaging (MRI) scan of the brain was arranged to look for any intracranial pathology for persistent headache and it revealed evidence of pituitary apoplexy with compression of optic chiasm and partial obliteration of bilateral cavernous sinuses. In view of MRI findings, a diagnosis of pituitary apoplexy with third cranial nerve involvement was considered the first differential diagnosis, and the patient was started on replacement hormones while temporarily withholding neostigmine. Following multidisciplinary input, it was decided to manage the patient conservatively. A repeat MRI brain was planned to assess the evolution which revealed resolution of pituitary apoplexy. Despite this, the patient continued to have ophthalmoplegia and fatigable partial ptosis. Pyridostigmine was restarted following which the patient fully recovered, confirming the diagnosis of ocular myasthenia gravis presenting concomitantly with pituitary apoplexy.

## Introduction

Pituitary apoplexy is a life-threatening condition caused by infarction or hemorrhage into a pituitary tumor and usually presents with sudden onset severe headache. It can be associated with ophthalmoplegia caused by cranial nerve palsies in one-quarter of patients as well [1]. The definitive diagnosis of pituitary apoplexy requires an MRI brain where a pituitary tumor with ischemic or hemorrhagic components inside it can be visualized [2].

Myasthenia gravis is an autoimmune neuromuscular condition mediated by acetylcholine receptor antibodies. When the clinical manifestations of myasthenia gravis are almost exclusively confined to ocular symptoms, it is referred to as ocular myasthenia gravis [3].

Even though both conditions can present with ophthalmoplegia, there are distinct clinical features and investigation findings that usually aid in differentiating between the two. The co-existence of pituitary tumors with myasthenia gravis has been reported occasionally in the literature [4]. However, co-presentation of pituitary apoplexy with ocular myasthenia gravis specifically has never been reported previously to our knowledge.

## Case presentation

A 53-year-old male patient presented with a sudden onset headache gradually worsening over two days with associated photophobia and phonophobia. He had no history of fever and the rest of the systemic inquiry was unremarkable except for mild diplopia and drooping of his left eyelid which he had noticed around the same time as the onset of headache. The patient had a past history of adrenal insufficiency diagnosed one year back. Even though further investigations had been planned to identify the cause of adrenal insufficiency, the patient had defaulted in follow-up. He did not have any significant past surgical history.

On examination, he was hemodynamically stable with a blood pressure of 116/76 and a pulse rate of 68 beats per minute. His Glasgow Coma Scale (GCS) was 15/15 and the rest of his cardiovascular, respiratory, and abdominal examination findings were unremarkable. On neurological examination, bilateral asymmetrical partial ptosis was observed with the left eye being more affected than the right eye along with mild fatiguability. There was associated left medial rectus palsy as well. However, there was no pupillary involvement and his visual fields were normal. The rest of the cranial nerve examination did not reveal any abnormalities. Apart from the above-mentioned neurological abnormalities, the rest of his neurological examination was unremarkable: specifically, he did not exhibit any muscle fatiguability in the rest of his body and Kernig's sign was negative as well.

Upon admission to the ward, he immediately underwent a non-contrast computed tomography (CT) scan of the brain which did not reveal any evidence of subarachnoid hemorrhage. Blood for basic investigations and cultures were taken but it did not reveal any evidence of infection.

Since the patient exhibited ocular fatiguability, a diagnosis of ocular myasthenia gravis was considered in the differential diagnoses, blood was sent for acetylcholine receptor antibodies (AchR Ab) and nerve conduction tests were arranged. Even though the AchR Ab test came as positive, the nerve conduction test did not show any decremental response supportive of myasthenia gravis. Based on the clinical suspicion and supportive serological findings, the patient was started on neostigmine, to which he showed a mild response only.

**Figure 1:**
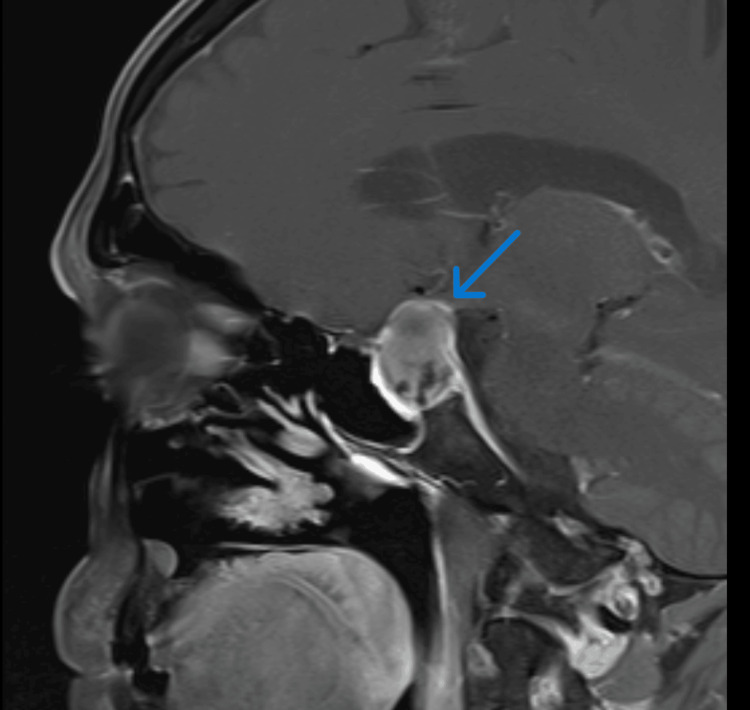
MRI showing enlarged pituitary gland with heterogenous internal signal intensities in keeping with pituitary apoplexy

**Figure 2:**
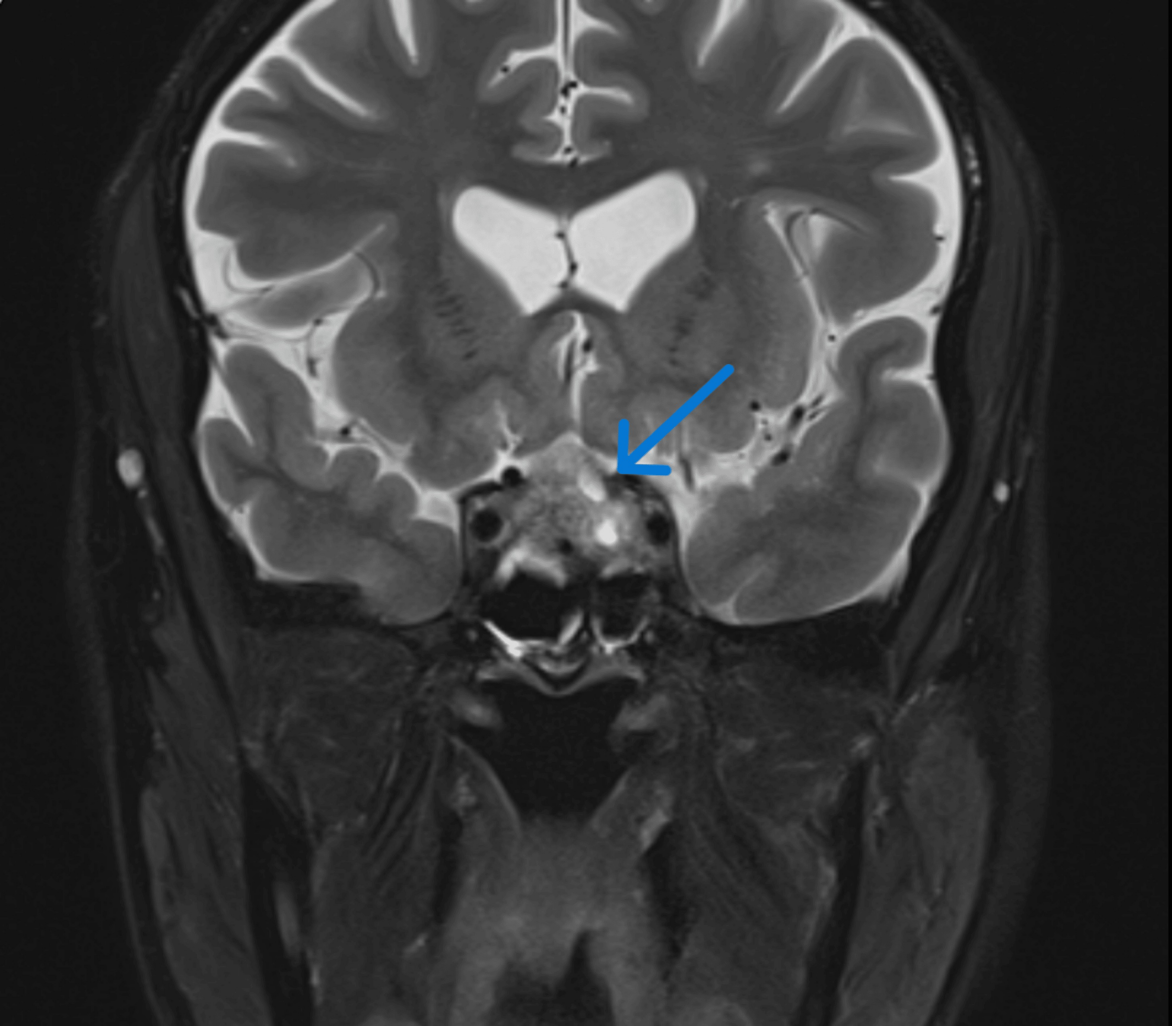
MRI (coronal view) showing pituitary apoplexy with compression of optic chiasm and partial obliteration of bilateral cavernous sinuses.

To exclude any intracranial pathology causing third cranial nerve palsy and to further evaluate the persisting headache, an MRI brain was also simultaneously arranged, which revealed evidence of pituitary apoplexy with compression of optic chiasm, partial obliteration of bilateral cavernous sinus with major displacement of optic chiasma (Figure 1, Figure 2).

In light of the MRI brain findings, a diagnosis of pituitary apoplexy causing third cranial nerve palsy was postulated. To assess the pituitary gland function, tests including the prolactin levels were sent, which are summarized in Table 1. A visual perimetry was also arranged but it did not reveal any visual field defects, particularly no bitemporal hemianopia was detected. In view of the associated hypopituitarism, the patient was started on replacement doses of IV hydrocortisone 50 mg every six hours, which was gradually tapered down and converted to oral hydrocortisone.

**Table 1:** Investigations performed to assess pituitary function TSH: Thyroid Stimulation Hormone; T3: Triiodothyronine; T4: Thyroxine; FSH: Follicular Stimulating Hormone; LH: Luteinizing Hormone; Na: Sodium; K: Potassium

Investigation	Patient Values	Reference range (for 53-year-old male)	Unit
Prolactin	1.49	2.1 – 17.7	ng/ml
9 a.m. cortisol	<27.6	101 – 535	nmol/l
TSH	1.349	0.35 – 4.94	µIU/ml
Free T3	2.32	1.58 – 3.91	pg/ml
Free T4	0.79	0.7 – 1.48	ng/dl
FSH	1.78	1.2 – 15.8	IU/l
LH	0.29	1.3 – 9.6	IU/l
Serum Na	126	136 - 145	mmol/l
Serum K	4	3.5 – 5.3	mmol/l

The patient was referred to the neurosurgical team for transsphenoidal resection of the pituitary gland; however, after multidisciplinary input from the neurosurgical, medical, and endocrinological teams, it was decided to manage the patient conservatively. Since the diagnosis was most likely to be pituitary apoplexy with involvement of the third cranial nerve, it was decided to continue the replacement doses of pituitary hormones and to withhold neostigmine for the time being.

**Figure 3:**
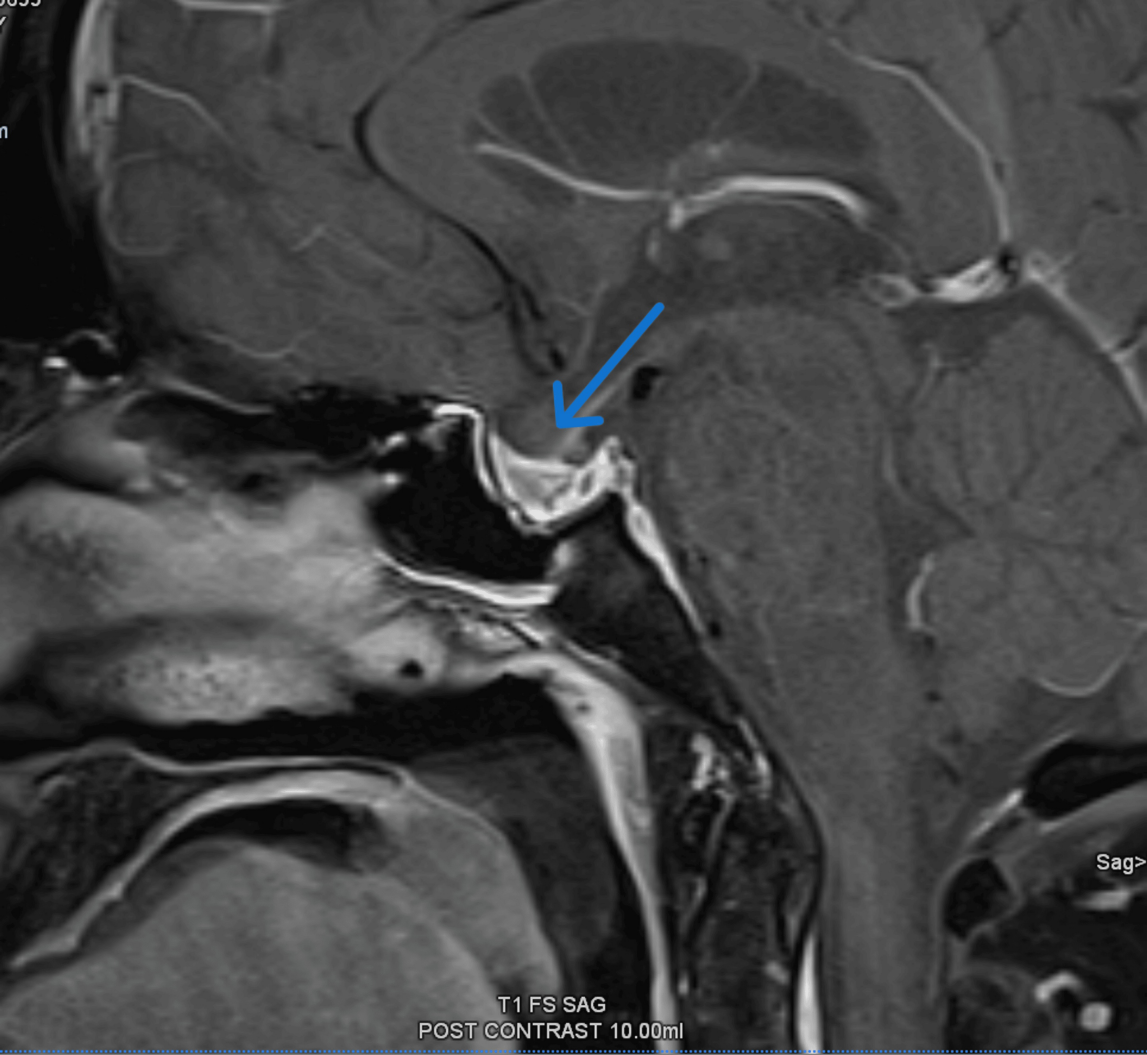
Heterogenous signal intensity with relatively normal size pituitary and thickened delayed enhancing pituitary stalk suggestive of residual changes following previous pituitary hemorrhage with no evidence of pituitary micro or macro adenoma

A follow-up MRI scan to assess the state of the pituitary apoplexy was arranged and it revealed a relatively normal-sized pituitary with residual changes following previous pituitary hemorrhage with no evidence of pituitary micro or macro adenoma. There was no evidence of compression of the optic chiasm or bilateral cavernous sinuses as well (Figure 3).

Despite the resolution of pituitary apoplexy on MRI and the patient becoming better with a resolution of headache, he continued to have partial ptosis with associated diplopia. At this stage, fatiguability could be easily demonstrated as well and with the positive clinical findings and the presence of AchR Ab, the diagnosis of ocular myasthenia gravis could be confirmed following which the patient was again started on pyridostigmine. He showed a significant response to the re-introduction of acetylcholinesterase inhibitors further reinforcing the diagnosis of ocular myasthenia gravis. A thoracic CT scan to look for any thymic pathology was also arranged but it did not reveal any abnormalities.

## Discussion

Pituitary apoplexy is hemorrhage or infarction of a pituitary tumor leading to rapid expansion of the tumor within the sella turcica. The classical presentation of pituitary apoplexy is a sudden onset severe headache commonly associated with visual impairment and ophthalmoplegia. The diagnosis can be confirmed by visualization of pituitary hemorrhage on brain imaging [5]. Ocular myasthenia gravis is an autoimmune condition caused by antibodies directed against acetylcholine receptors in the neuromuscular junction. In contrast to myasthenia gravis, in ocular myasthenia only the ocular muscles are exclusively affected leading to symptoms like ptosis and diplopia [3].

In the present case, the diagnosis dilemma was between the third cranial nerve palsy caused by pituitary apoplexy vs ocular myasthenia gravis. Even though within the clinical context myasthenia gravis was considered the first differential diagnosis, with the finding of pituitary apoplexy on MRI with evidence of partial obliteration of bilateral cavernous sinuses, third cranial nerve palsy caused by pituitary apoplexy itself was also considered. Third cranial nerve involvement caused by pituitary apoplexy has been reported in the literature and there are several postulated mechanisms that lead to cranial nerve palsies in pituitary apoplexy. One of them is oculomotor palsy caused by direct involvement of the nerve due to tumor invasion [6]. Other mechanisms like compression of the vasa nervorum leading to reduced blood supply to the nerve, surrounding edema following pituitary apoplexy, or ischemic infarction of the tumor causing compression have also been brought forward in the literature [6,7].

The findings in favor of myasthenia gravis were the mild fatiguability and the lack of pupillary involvement in the initial examination. This was further reinforced by the positive AchR Ab status and the patient’s clinical response to the neostigmine treatment. However, the lack of decremental response in nerve conduction studies stood against a diagnosis of myasthenia gravis. In addition, the finding of pituitary apoplexy with evidence of partial obliteration of bilateral cavernous sinuses in the MRI made the diagnosis of ocular myasthenia gravis further unlikely. Since the contemporaneous occurrence of myasthenia gravis with pituitary apoplexy was very unlikely and a causal relationship between the two has never been mentioned in the literature to our knowledge, the diagnosis of third cranial nerve palsy caused by pituitary apoplexy itself was considered and the treatment with neostigmine was temporarily withheld. However, the fact that the patient’s symptoms continuously worsened with the omission of neostigmine treatment even after resolution of pituitary apoplexy was observed in the MRI, was again in favor of ocular myasthenia gravis. The diagnosis of ocular myasthenia gravis was fully confirmed with the full resolution of symptoms after the reintroduction of pyridostigmine.

Cessation of neostigmine and initiation of IV hydrocortisone for hypopituitarism both were done around the same time period following which transient worsening of the ocular symptoms was also observed. Transient worsening of myasthenia gravis symptoms with the initiation of steroids is a known phenomenon and this could also have contributed to the worsening of diplopia [8].

In the literature, there are few case reports where myasthenia gravis has been associated with pituitary adenomas; however, to our knowledge, a case of ocular myasthenia gravis occurring alongside pituitary apoplexy has never been reported in the literature. There are case reports where myasthenia gravis has been associated with pituitary adenomas [4], commonly prolactin-secreting adenomas, non-functioning adenomas, and growth hormone-secreting adenomas, but never presenting simultaneously with pituitary apoplexy. In a case report of myasthenia gravis imitating pituitary apoplexy by Zoli et al., a patient with macroprolactinoma presented with diplopia, ptosis, and acute onset headache with rapid clinical deterioration suggestive of pituitary apoplexy and underwent immediate surgery only to discover that there was no pituitary hemorrhage and that the symptoms have all along been due to myasthenia gravis [9].

The presentation of myasthenia gravis with pituitary apoplexy could be a mere coincidence, but a causal relationship cannot altogether be excluded. The patient had a history of adrenal insufficiency for which he had defaulted on follow-up. So, the possibility of a pre-existing pituitary tumor, similar to the other case reports published in the literature, cannot altogether be excluded as well. Nevertheless, the contemporaneous occurrence of these seemingly unrelated two conditions poses a mighty diagnostic difficulty.

The mainstay of management of ocular myasthenia gravis includes acetylcholine esterase inhibitors, immunosuppressive agents, and steroid-sparing agents like azathioprine and mycophenolate mofetil. In the presence of associated thymic hyperplasia or thymoma, thymectomy can also be done. Other surgical options include correction of strabismus and ptosis repair [3].

The management options for pituitary apoplexy are either conservative or surgical management. The choice between the two is individualized. Usually, the presence of neuro-ophthalmic complications, impairment of consciousness, and deteriorating neurological signs warrant surgery in the absence of other contraindications. Nevertheless, a proper comparison between the two has not been done and the available evidence suggests that the endocrinological outcomes are similar in both groups [5].

## Conclusions

Pituitary apoplexy is a life-threatening condition which usually presents with sudden onset severe headache. It can rarely present with ophthalmoplegia when the cranial nerves, commonly the third cranial nerve, are involved. Meanwhile, ocular myasthenia gravis is a variant of myasthenia where ocular muscles are exclusively affected. Ophthalmoplegia presenting with clinical evidence supportive of both aforementioned conditions can lead to a diagnostic dilemma. Additionally, contemporaneous presentation of ocular myasthenia gravis with pituitary apoplexy has never been reported in the literature and a causative relationship between the two is yet to be established.
